# The Effect of Resveratrol on the Composition and State of Lipids and the Activity of Phospholipase A_2_ During the Excitation and Regeneration of Somatic Nerves

**DOI:** 10.3389/fphys.2019.00384

**Published:** 2019-04-18

**Authors:** Victor Vasilevich Revin, Sergey Ivanovich Pinyaev, Marina Vladimirovna Parchaykina, Elvira Sergeevna Revina, Georgiy Vladimirovich Maksimov, Tatyana Pavlovna Kuzmenko

**Affiliations:** ^1^Department of Biotechnology, Bioengineering and Biochemistry, National Research Ogarev Mordovia State University, Saransk, Russia; ^2^Department of Biophysics, Moscow State University, Moscow, Russia

**Keywords:** myelinated nerve fibers, rhythmic excitation, nerve intersection, resveratrol, phospholipids

## Abstract

It has been shown that in the somatic nerve’s lipids, both during excitation and transection, changes occur with the composition of individual phospholipids and in phospholipids fatty acids, which changes the phase state of the myelin and nerve fiber axolemma lipid bilayer. A main contribution in the nerve degenerative processes is dependent on the composition phospholipid’s fatty acid changes during the activation of both Ca^2+^-dependent and Ca^2+^-independent phospholipase A_2_ forms. At the same time, we studded changes in phosphoinisitol (PI) and diacylglycerol (DAG), which depend on the phosphoinositide cycle function during nerve excitation and degeneration processes. It was found that myelin lipids and nerve fiber axolemmas are involved not only in the functioning of the peripheral nerves, but also the pathological processes underlying deep functional and structural disorders. The effect of resveratrol on regeneration processes in the damaged rat sciatic nerve has also been investigated.

## Introduction

In recent years, a significant number of studies have shown that the lipid bilayer defines and, in some cases, plays a major role in the functioning of receptors, ion-transporting systems, and nearly all the structures localized in the membrane (Revin et al., 1996, 2006, 2012; Fernandis and Wenk, 2007; Ayala et al., 2014; Ge and Liu, 2016; Pöyry and Vattulainen, 2016).

Considering the fact that the bilayer is in different phase states and depends on the composition of individual polar regions in charge-carrying phospholipids and fatty acids forming their hydrophobic areas, it can be assumed that by studying their composition and condition, their participation in the processes of physiological functioning and the primary mechanisms of development of pathological processes can be evaluated (Revin et al., 2006, 2012; Ge and Liu, 2016; Pöyry and Vattulainen, 2016).

There is a interest in the study of their role for neurobiology in direction of the excitation conduction of and the damaged somatic nerves regeneration.

One of the classic models for the above processes studes is somatic nerves. The aim of our study was to investigate the composition and condition of lipids changes during the somatic nerves excitation and damage. The enzyme phospholipase A_2_, especially Ca^2+^-dependent phospholipase A_2_, plays an important role in the study of lipid composition (O’Donoghue et al., 2011; Nardicchi et al., 2014).

Another aim of this work is to show the differences of nerve parts (proximal) and the distal during the regeneration. This series of experiments will estimate the contribution of central innervation to the somatic nerves regeneration.

Despite the modern medicine successes in the restoration of damaged peripheral nerves investigation, the issue of complete nerve regeneration has not been resolved. This fact encourages the search for new methods and physiologically active substances that can stimulate regeneration processes in affected nerves (Vance et al., 2000; Michalski et al., 2008; More et al., 2012; Grinsell and Keating, 2014; Chu et al., 2017). Resveratrol is one of the most promising substances of natural origin and has antioxidant, anti-inflammatory and neuroprotective properties (Institute of Laboratory Animal Resources (US), 1985; Pallàs et al., 2009; Namsi et al., 2018).

According these data is to study the resveratrol effect of on regeneration processes in damaged nerves.

## Materials and Methods

As the object of study was the sciatic nerves of adult white Wistar rats (weighing 200–250 g) was used. Experiments using animals were carried out in accordance with the guidelines for the treatment, maintenance and use of laboratory animals (Institute of Laboratory Animal Resources (US), 1985). The isolated nerve bioelectrical activity was recorded extracellularly with the following electrostimulation parameters: amplitude 1.5 V, duration 0.3 ms, frequency stimulation 100 imp/s.

The experimental design included several variants. In the first series of experiments, we compared the nerve without stimulation (rest, control) and nerves that were stimulated with an alternating electric current of 100 imp/s for 5 min. In the second experimental series, a model of pathology was created when the sciatic nerve of the rat was cuted: in one the animals of one group the sciatic nerves was cuted, and sutured; in the other animals group, *trans-*resveratrol (Shaanxi Honghao Bio-Tech Inc., China) at 100 μl, 0.1 M was perfused daily in the cutting area (in the broad medial thigh muscle). The intact rat sciatic nerve served as the control. The proximal and distal parts of the sciatic nerves as well as the control nerves were investigated after 7 days. All work was carried out in accordance with the Declaration of Helsinki (1964).

The lipid extraction from the nerve was carried out according to the Bligh-Dyer method (Bligh and Dyer, 1959). For the analysis of phospholipids (PL), high-performance thin layer chromatography with a solvent system of chloroform/methanol/water/ammonia (60/34/4/2) (Reich and Schibli, 2004; Handloser et al., 2008) and chloroform/methanol/glacial acetic acid/water (60/50/1/4) (Evans and Findlay, 1987) was used. For the separation of diacylglycerol (DAG), a heptane/diethyl ether/glacial acetic acid solvent system (60/40/2 by volume) was used (Evans and Findlay, 1987). Lipid fractions were identified using specific markers (Supelco, UnitedStates). The lipids contents was controlled by a densitometric method using an automated complex CAMAG TLC Scanner 4 (Switzerland) (Revin et al., 2016). The content of PL was calculated using the ratio of the inorganic phosphorus content of individual PL fractions to the total inorganic phosphorus content of all PL fractions.

For the fatty acid contents in the individual lipid fractions analysis, methylation of fatty acids (FA) was carried out using the Morrison and Smith method (Morrison and Smith, 1964). The separation of methyl esters of FA was carried out on a SHIMADZU GC-2010Plus AF gas chromatograph (Japan) (Revin et al., 2016).

To determine the nerve homogenate phospholipase A_2_ activity, the changes of the phosphatidylcholine free fatty acids (FFA) content during hydrolysis was estimated using gas chromatography. The incubation medium for determining the activity of Ca^2+^-dependent phospholipase A_2_ contained: 10 mM Tris, 0.05 M NaCl, 5 mM CaCl_2_, and 0.5% Triton X-100 and pH 8. The incubation medium for controlling the Ca^2+^-independent phospholipase A_2_ activity contained: 10 mM Tris, 0.05 M NaCl, 1 mM EGTA (ethylene glycol tetraacetic acid), and 0.5% Triton X-100, pH of 8 (Vladimirov Yu, 1998). The specific activity of PL A_2_ was expressed in μg FA formed for 1 h per 1 mg protein. The protein content was determined by using the Lowry method (Lowry et al., 1951).

Phosphatidylcholine was isolated from egg yolk and purified by high-performance thin layer chromatography using a solvent system of chloroform/methanol/acetone/glacial acetic acid/water (40/13/15/12/8, by volume) (Evans and Findlay, 1987).

The determination of the temperature of the lipid phase transition was carried out using a module DSC822e differential scanning calorimeter (Switzerland).

The statistical processing of the data was carried out with the help of Statistica 10 software.

## Results and Discussion

It has been known that lipids participate in the functions of cell membranes that are related to their composition and high exchange rate, and various fatty acids determines the bilayer physical state and the ability to oxidize (Revin et al., 2006). Using layer chromatography, we investigated the change in lipid composition of the rat nerve after stimulation, damage, and resveratrol action.

**FIGURE 1:**
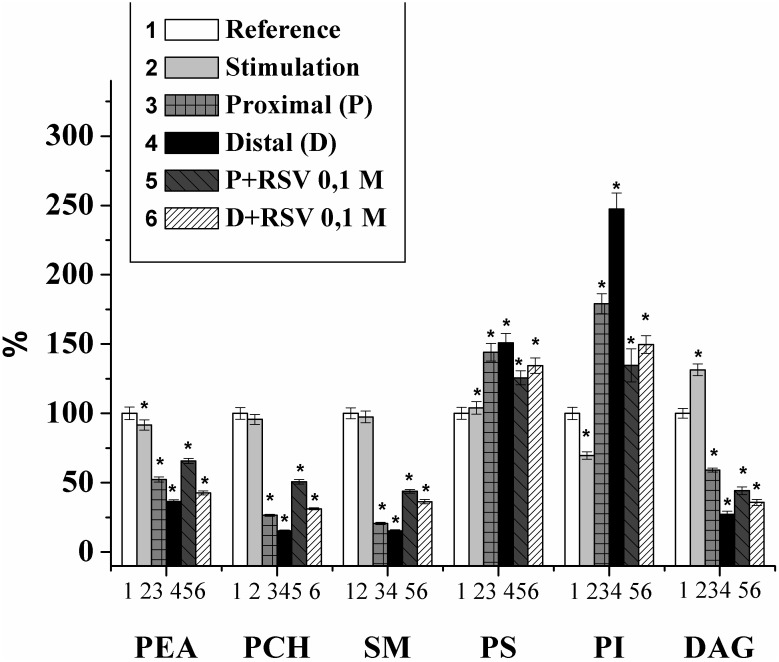
Changes of the individual lipid fraction contents during the stimulation and damage of the rat myelin nerve and the action of resveratrol (RSV, 10^-1^ M) (% of control); ^∗^significance of difference in relation to control, *p* < 0.05.

From the studies on the changes in individual phospholipid compositions in the rat nerve, it was eslished that the content of phosphatidylethanolamine (PEA) in the nerve (of the first experimental group) was 236.56 ± 10.81 μg P PEA/mg P PL, that of phosphatidylcholine (PCH) was 128.53 ± 5.4 μg P PCH/mg P PL, that of phosphatidylserine (PS) was 120.01 ± 5.21 μg P PS/mg P PL, that of sphingomyelin (SM) was 137.39 ± 5.4 μg P SM/mg P PL, that of phosphatidylinositol (PI) was 50.36 ± 2.26 μg P PI/mg P PL, and that of diacylglycerol (DAG) was 14.25 ± 0.54 μg fatty acid/mg common lipids. During the electrical stimulation of the nerve (100 imp/s for 5 min), the content of PI and PEA decreased by 30.3 and 8.4%, respectively, and the content of DAG increased by 31.4% compared to that of the control. In the remaining fractions studied, no significant change was detected (Figure 1).

Thus, it was shown that nerve excitation leads to a decrease in the PI fraction and an increase in DAG, which is the product of phospholipid cleavage by phospholipase C and plays a key role in the regulation of the activities of phospholipase A_2_ and protein kinase C and the influx of Ca^2+^ (Treuheit et al., 2016). In addition, DAG can activate protein kinase C and is involved in signaling from the receptor of the excited cell surface to the performing proteins and phospholipase A_2_ (Pungerèar and Križaj, 2007).

Previously, it was found that the lipid composition in damaged nerves is significantly changed (Revin et al., 1996, 2006, 2012, 2015; Isakina et al., 2015). Therefore, for the control, we used the experiments where the changes in the PL composition were studied after the nerve cutting. It was found that after the 7th day of the injury, the amounts of PEA, PCH, SM, and DAG in the proximal part of the nerve decreased by 47.7%, 73.5%, 79.4%, and 40.8%, respectively, and that the amounts of PS and PI increased by 44.1% and 73.9%, respectively. In the distal part of the nerve, the contents of PEA, PCH, SM, and DAG decreased by 63.6%, 84.6%, 84.7%, and 72.7%, respectively, and the contents of PS and PI increased by 50.9% and 147.6%, respectively, compared with those of the control on the 7th day of observation (Figure 1).

The changes of PI and DAG contents in the damaged nerve are consistent with our previous studies indicating an increase of PI and a decrease in DAG during the inactivation of the phosphoinositide-specific phospholipase C (Ramassamy, 2006). At the same time, an PI increase leads to an growth in the density of the membrane charged groups, binding the Ca^2+^ (Revin et al., 1996). It should be noted that in the distal nerve segment, changes in the content of individual lipids occur much more intensively than in the proximal segment.

Thus, after the nerve cutting, profound changes occurred in both the distal and proximal parts of the damaged nerve. We note that the changes in the distal part of the nerve were more pronounced, which is possibly connected to the disappearance of the central nervous system innervation, the blocking of axonal transport, and the increase in the rate of nerve degeneration (Arkhipova et al., 2009).

In the next series of experiments, the effect of resveratrol on the lipid composition of the myelin nerve was investigated. It was found that when an animal is injected with resveratrol (0.1 M), increases in the lipid content of PEA, PCH, and SM in the nerve proximal part were observed to be 25.6%, 91.1%, and 112.9%, respectively, in comparison with that in the cut nerve not treated by the drug. In the distal segment of the neural conductor, the levels of PEA, PCH and SM increased by 17%, 103.1%, and 71.7%, respectively, compared with those in the injured nerve. In this version of the experiment, the contents of PS and DAG decreased by 12.8 and 25.3%, respectively, at the proximal end of the nerve compared to the those at the injured nerve. In the distal segment of the sciatic nerve of the rat that received the 0.1 M resveratrol injection, the PS content was reduced by 11%, and the DAG level increased by 31.1%, compared to those in the injured nerve. The level of PI against the background value of that under resveratrol action decreased by 26.2% at the proximal end of the nerve and 41.86% in the distal nerve segment relative to the level in the damaged nerve (Figure 1).

The stabilizing effect of resveratrol on the nerve fiber membranes phospholipid composition of can be explained by the fact that the resveratrol molecule is lipophilic. Transport and interaction with resveratrol occur as a result of its entry into free volumes, the presence of which is determined by mobile structural defects, namely, kinks. In addition, resveratrol can be transported through lipid rafts (Tsuchiya, 2001), which facilitate the penetration of large amounts of molecules; hence, resveratrol has a greater effect on the damaged nerve fiber. By changing the lipid fraction-to-membrane ratio, the lipid packing of the cell membrane bilayer is changed accordingly. Resveratrol may play a key role in membrane processes, namely, the functioning of membrane proteins that regulate cellular signaling (Delmas and Lin, 2011). Therefore, RSV can be involved in the mechanism of regulation of the activity of transmembrane proteins, such as G-proteins, phospholipase C and protein kinase C, there by participating in signal transduction and influencing cellular proliferation and susceptibility to apoptosis by regulating the fluidity of the cell membrane (García-García et al., 1999; Brittes et al., 2010).

It has been known that the activation of phosphatidylinositol-3-kinase initiates PI phosphorylation and increases the concentrations of phosphatidylinositol-3-phosphate, phosphatidylinositol-3,4-diphosphate and phosphatidylinositol-3,4,5-triphosphate in the cell. In turn, phosphatidylinositol-3-phosphate activates the serine/threonine kinase, Akt (Kasdallah-Grissa et al., 2006). Thus, the stabilizing effects of resveratrol on the lipid composition of the damaged nerve membrane and its possible involvement in cellular signaling through the membranes and on lipid fluidity confirms its active influence on regeneration processes in damaged nerves.

Interest in the study of the fatty acid composition of nerve membranes is explained by their important role in cells functions. In addition, the unsaturated fatty acids in the membrane control the phase states and make them sensitive to various effects, which can change the activities of individual enzyme systems and cell function.

We studied the fatty acid composition of sciatic nerve phospholipids during excitation after its injury and the action of resveratrol. In the fatty acids of the individual fractions of lipids, 21 fatty acids were found: decane (10: 0), neodecanoic (11: 0), lauric (12: 0), tridecane (13: 0), myristic (14: 0), myristoleic (14: 1), pentadecanoic (15: 0), *cis-*10-pentadecane (15: 1), palmitic (16: 0), palmitoleic (16: 1), stearic (18: 0), elaidic (18: 1n9t), oleic (18: 1n9c), linoleic (18: 2n6c), linolenic (18: 3n3), arachine (20: 0), *cis-*11,14-eicosadiene (20: 2), *cis-*8,11,14-eicosatrienic (20: 3n3), arachidonic (20: 4n6), *cis-*13, 16-docosadiene (22: 2), and lignoceric (24: 0).

**FIGURE 2:**
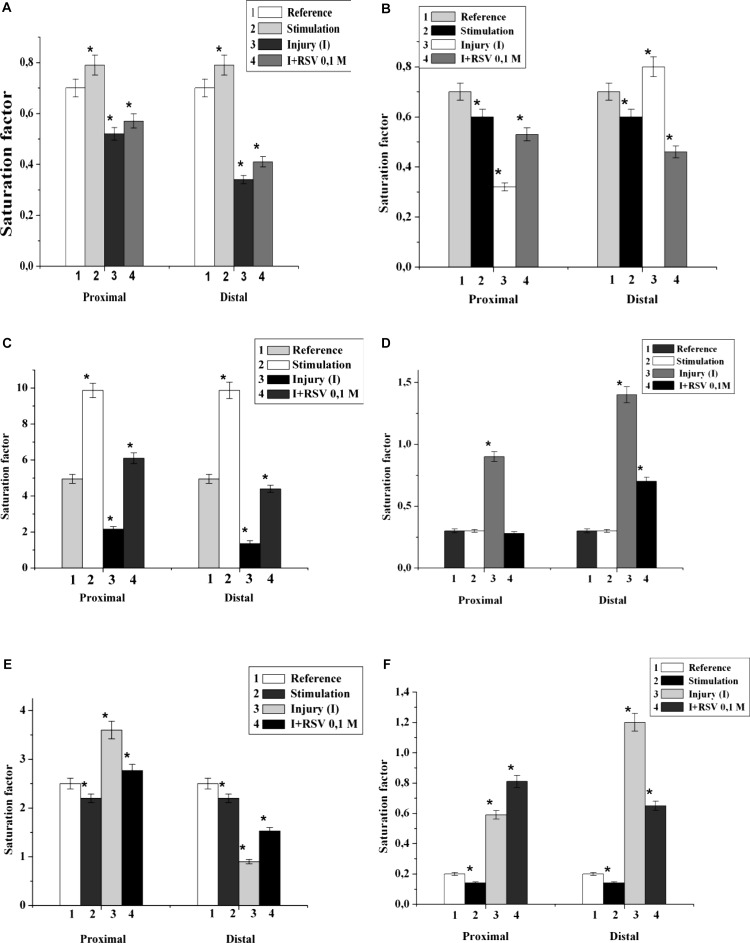
The effect of nerve cutting on the saturation factor of: **(A)** phosphatidylethanolamine, **(B)** sphingomyelin, **(C)** diacylglycerin, **(D)** phosphatidylserine, **(E)** phosphatidylcholine, and **(F)** phosphatidylinositol in the proximal and distal nerve sections. I – injury, RSV – resveratrol, ^∗^significance of difference in relation to the control, *p* < 0.05.

It has been found that 5 min nerve stimulation leads to a decrease in the PI saturation factor by 1.4 times and an increase in DAG by 2.0 times in comparison with those of the control (Figure 2C,F). It is important that the maximum changes were found in the fractions of palmitic, stearic, oleic, linoleic and arachidonic acid. It has been established that the FA composition of lipids in the proximal end of the nerve after injury changes significantly. Thus, in the fractions of PEA, SM, and DAG after nerve cutting, the saturation factor decreased by 1.4, 2.1, and 10.0 times, respectively, compared with that of the control (Figure 2A–C). The contents of saturated FA in the palmitic and stearic fractions decreased by an average of 1.5 times, and the levels of unsaturated FA (oleic, linolenic, and arachidonic) increased by an average of 1.7 times.

In the investigation of the fractions of PS, PCH, and PI, it was found that the saturation coefficients were increased by 3.0, 1.5, and 3.1 times, respectively (Figure 2D–F). At the distal end of the nerve, after the 7th day of the experiment, the saturation coefficient decreased by 7.0, 2.8, 3.5, and 10.0 times in PEA, PCH, SM, and DAG, respectively, compared to that of the control. In the PS and PI fractions, the saturation coefficient increased by 6.0 and 8.0 times, respectively (Figure 2D,F).

It was found that the injection of resveratrol increased the saturation factors of PEA, SM, DAG and PI by 1.2, 1.7, 12.4, and 1.4 times, respectively, compared with those of the cut nerve (Figure 2A,B,C,F); in addition, the saturation factor of PS decreased by 1.8 times compared to that of the cut nerve (Figure 2D). During the resveratrol action, the saturation factors of PEA, SM, and DAG at the distal part of the nerve increased by 1.5, 1.1, and 10.0 times, respectively, compared with that of the cut nerve. At these conditions, the saturation factors of PS and PI decreased by 1.6 and 1.3 times, respectively. Thus, the nerve cutting was accompanied by a redistribution of the individual lipid fatty acids of each nerve component, which indicates processes occurring in the hydrophobic region of the membrane. The resveratrol action possibly stabilizes the lipid fatty acid composition and restores the nerve membranes.

**FIGURE 3:**
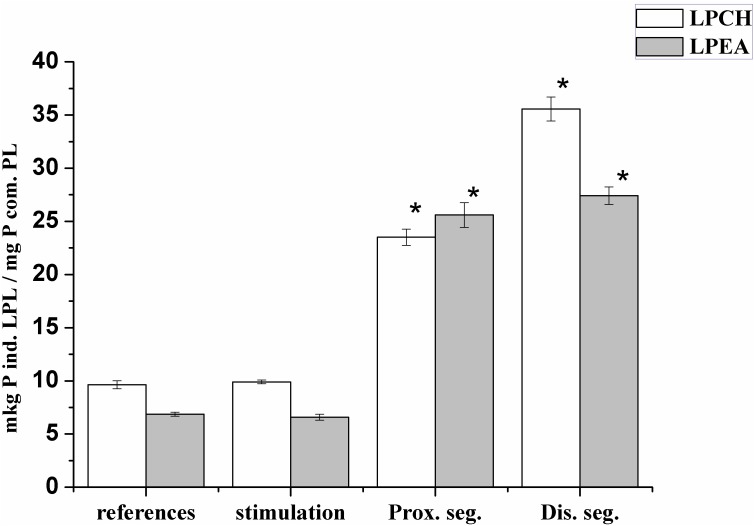
Changes in the levels of lysophosphatidylcholine (LPCH) and lysophosphatidylethanolamine (LPEA) in the myelin rat nerve during stimulation and after nerve cutting; ^∗^the significance of the difference in relation to control, *p* < 0.05.

**FIGURE 4:**
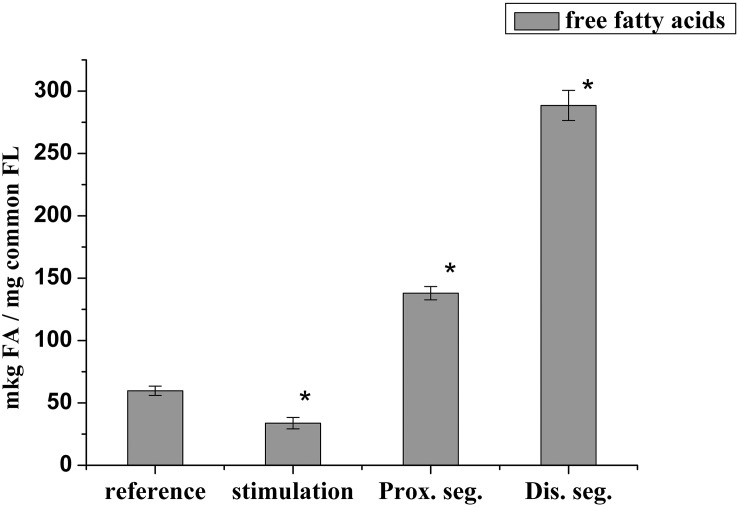
Changes in the content of free fatty acids during the stimulation of the rat myelin nerve and after nerve cutting; ^∗^significance of the difference with respect to control, *p* < 0.05.

It has been known that lysophospholipids (LPL) and FFA form during the activation of the lipolytic enzyme, phospholipase A_2_, which takes place in many pathological processes. During this experiment, it was found that the lysophosphatidylcholine (LPCH), lysophosphatidylethanolamine (LPEA), and FFA contents in rat myelin nerve were 9.64 μg P LPCH/mg P PL, 6.86 μg P LPEA/mg P PL, and 59.7 μg FA/mg PL, respectively. Nerve excitation during 5 min led to a decrease in the level of FFA by 43.3% (*p* < 0.05), but the phospholipids lysoforms contents were not changed. During the 7th day of nerve cutting, the contents of LPCH, LPEA and FFA increased (by 2.4, 3.7, and 2.3 times, respectively) at the proximal site of the nerve. In the distal site, the contents of LPCH and LPEA increased by 3.7 and 5.5 times (*p* < 0.05), respectively, relative to that of the control, and the level of FFA increased by 3.8 times (*p* < 0.05) compared to that of the intact nerve (Figure 3, 4).

It is likely that the accumulation of lysophospholipids during nerve damage is due to an increase in the activity of PL A_2_, which catalyzes the hydrolysis of phospholipids of polyunsaturated FA (Isakina et al., 2015). This has been evidenced by data on the participation of PL A_2_ in the process of early myelin degradation during Waller’ degeneration of rat and mouse nerves and frog sciatic nerves (Nardicchi et al., 2014; Revin et al., 2015). Not only the lysophospholipids but also the FFA have a detergent effect and are powerful regulators of physiological and biochemical processes. Therefore, the increases in LPL and FFA after 7 days trauma are a marker of the development of degeneration processes in each site of the damaged nerve.

**FIGURE 5:**
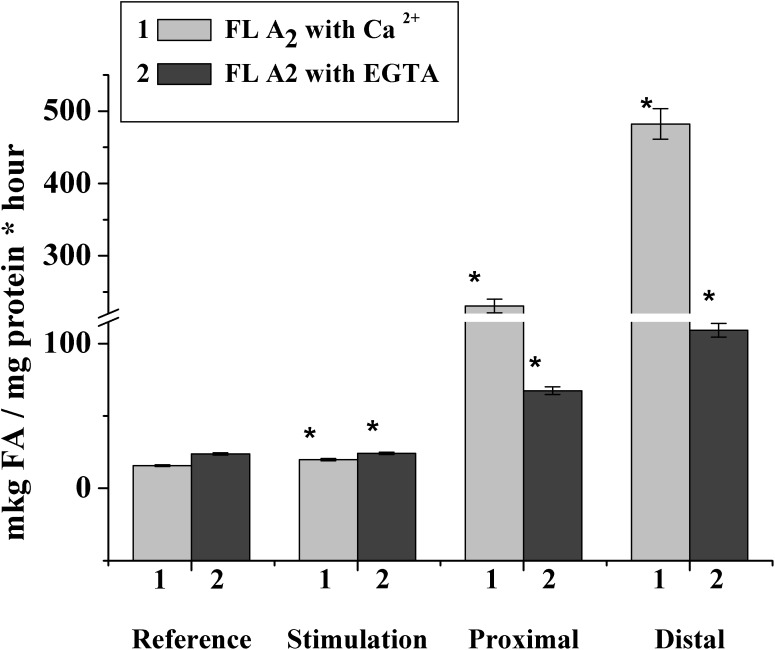
Changes in phospholipase A_2_ activity after incubation of the rat myelin nerve in Ca^2+^- or EGTA-containing medium during the electric stimulation and damage of the nerve; ^∗^significance of difference with respect to control, *p* < 0.05.

To confirm this hypothesis, we studied the phospholipase activity during the nerve stimulation and after nerve cutting. It was found that in the rat myelin nerve, the phospholipase A_2_ activity was 15.0 μg of fatty acids/mg protein and increased by 26.2% after the nerve was stimulated for 5 min; after the 7th day of nerve cutting, the activity increased by 14.7 times in the proximal site and 30.7 times in the distal site (Figure 5).

It has been known that the family of intracellular phospholipase A_2_ includes Ca^2+^-dependent and Ca^2+^-independent phospholipase A_2_, which change activity during the myelin sheath damage at Waller’s degeneration (Ames et al., 1993; Ramassamy, 2006). Based on this connection, we investigated changes in the activities of both Ca^2+^-dependent and Ca^2+^-independent phospholipase A_2_.

It was found that during the nerve excitation, changes in enzyme activity without Ca^2+^ (addition of an EGTA in the incubation medium to record the activity of Ca^2+^-independent phospholipase A_2_) were not observed. Nerve injection led to an increase in the activity of Ca^2+^-independent phospholipase A_2_ in the proximal and distal nerve sites by 2.8 and 4.6 times, respectively (Figure 5).

Thus, excitation along the nerve is accompanied by a change in the activity of Ca^2+^-dependent, but not Ca^2+^-dependent, phospholipase A_2_, although a significant increase in the enzymatic activity of both forms of phospholipase A_2_ is observed when the nerve is damaged.

The most important thing should also be noted that during the nerve excitation and damage we found the phosphoinositide’s and DAG content changes. It is known that the metabolism of PI and their metabolic products is associated with the regulation of number of the intracellular processes (Raben and Barber, 2017).

First, DAG can trigger several processes related to membrane Ca^2^^+^ transport (Land and Rubin, 2017) and the triphosphoinositol stimulation to release Ca^2+^ from intracellular depots (Streb et al., 1983). All this initiates the rapid and short-term increase in free Ca^2+^ inside the myelin nerve, and might activate a large number of enzymatic reactions it is possible that participates in the activation of phospholipase A_2_, which we observed in our experiments.

**FIGURE 6:**
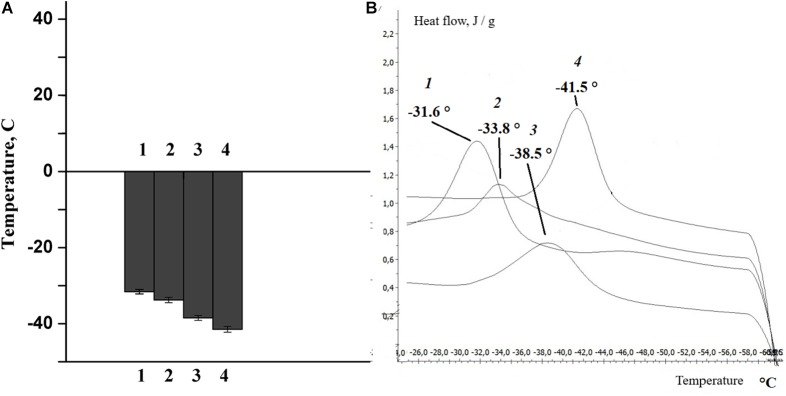
**(A)** Histogram, **(B)** differential scanning calorimetry curves of lipids isolated from the sciatic nerve of the rat: control (1), after nerve stimulation (2), after nerve cutting at the proximal site (3), and at the distal site (4).

The change in lipid composition is one of the mechanisms by which the physico-chemical state of the myelin lipid bilayer regulates the nerve fiber phospholipid composition (Rajapaksha et al., 2015; Treuheit et al., 2016). In the next experiments, changes in the phase state of the extracted lipids were studied after rat nerve excitation and damage. It was found that the temperature of the extracted lipid phase of the myelinated nerve of the rat transitioned from -31.6°C (at rest) to -33.8°C (after nerve excitation), but nerve cutting led to a significant decrease in the temperature of the extracted lipid phase during the 7th day of the experiment: to -38.5°C at the proximal region of the nerve and to -41.5°C in the distal region of the nerve (Figure 6).

It is well known that phase transition plays an important role in changing the density and order of the membrane phospholipids, the thickness of the membrane lipid bilayer and the formation of non-specific permeability for calcium ions. This is confirmed by the results of our own studies, which show that the structural alteration after the activation of phospholipases (the lipid composition of the bilayer) and the corresponding change in the physicochemical properties of the lipids are accompanied by a change in myelin properties during normal nerve function and pathology.

## Conclusion

It was established that both the nerve excitation conduction and the transection of somatic nerves lead to profound changes in the lipid composition. Not only the number of phospholipids, but also the phospholipid fatty acid composition is changing. The key factors responsible for the detected changes are phospholipase A_2_ and phospholipase C activities. In the first place of the nerve fiber degenerative processes were both Ca ^2+^-dependent and Ca ^2+^-independent phospholipase A_2_ activation in the fiber’s distal part, which associated with of the axonal transport blocking.

Another factor affecting the nerve excitability and the degenerative processes dynamics is the activation of the phosphoinositide’s metabolism of Revin et al. (1996, 2006). The differences and directions of changes in the amount of PI and DAG that we found during excitation and at the degeneration indicate disorders in the somatic nerves phosphoinositides metabolism and enzyme systems responsible for both the phosphorylation of individual forms of PI and its hydrolysis.

One of the mechanisms of action of resveratrol on the nerve regeneration process is associated with its antioxidant ability, due to the leveling of the content of hydroxyl, superoxide and other radicals. All this is manifested in the protection of cell membranes from lipid peroxidation and DNA damage (Kovacic and Somanathan, 2010; Huang et al., 2014). Probably, changes in the content of PI and DAG, as well as the redistribution of fatty acids of various lipid fractions have an effect on the protein lipid relationships and the orderliness of fatty acids (viscosity) in the myelin and axolemma nerve fiber. All this might change the activity of transmembrane proteins, such as phospholipase C and protein kinase C, and the process of cellular signaling, including the phosphoinositide-3-kinase pathway.

## Ethics Statement

All animals for this study were placed in the Animal Care Facility of Ogarev Mordovia State University under standard conditions with free access to water and food. All animal procedures were performed according to a protocol approved by the Institutional Animal Care and Use Committees of the Medical Institute of Ogarev Mordovia State University (Ethics Committee protocol No. 50 from 20.05.2017).

## Author Contributions

VR conceived and designed the study. SP, MP, and TK designed and performed experiments. SP and MP wrote the first draft of the manuscript. VR, SP, MP, GM, and ER wrote sections of the manuscript. All authors contributed to manuscript revision, read and approved the submitted version.

## Conflict of Interest Statement

The authors declare that the research was conducted in the absence of any commercial or financial relationships that could be construed as a potential conflict of interest.
